# Patient-level predictors of temporal regularity of primary care visits

**DOI:** 10.1186/s12913-023-09486-5

**Published:** 2023-05-08

**Authors:** Adam J. Rose, Wiessam Abu Ahmad, Faige Spolter, Maram Khazen, Avivit Golan-Cohen, Shlomo Vinker, Ilan Green, Ariel Israel, Eugene Merzon

**Affiliations:** 1 Braun School of Public Health and Community Medicine, Faculty of Medicine, Hebrew University of Jerusalem, Ein Kerem Campus, Jerusalem, Israel; 2 Leumit Health Services, Research Institute, Tel Aviv, Israel; 3 Sackler School of Medicine, Tel Aviv, Israel; 4 Adelson School of Medicine, Ariel University, Ariel, Israel

**Keywords:** Quality of health care, Temporal regularity of primary care, Case mix adjustment, Primary care

## Abstract

**Background:**

Patients with chronic diseases should meet with their primary care doctor regularly to facilitate proactive care. Little is known about what factors are associated with more regular follow-up.

**Methods:**

We studied 70,095 patients age 40 + with one of three chronic conditions (diabetes mellitus, heart failure, chronic obstructive pulmonary disease), cared for by Leumit Health Services, an Israeli health maintenance organization. Patients were divided into the quintile with the least temporally regular care (i.e., the most irregular intervals between visits) vs. the other four quintiles. We examined patient-level predictors of being in the least-temporally-regular quintile. We calculated the risk-adjusted regularity of care at 239 LHS clinics with at least 30 patients. For each clinic, compared the number of patients with the least temporally regular care with the number predicted to be in this group based on patient characteristics.

**Results:**

Compared to older patients, younger patients (age 40–49), were more likely to be in the least-temporally-regular group. For example, age 70–79 had an adjusted odds ratio (AOR) of 0.82 compared to age 40–49 (*p* < 0.001 for all findings discussed here). Males were more likely to be in the least-regular group (AOR 1.18). Patients with previous myocardial infarction (AOR 1.07), atrial fibrillation (AOR 1.08), and current smokers (AOR 1.12) were more likely to have an irregular pattern of care. In contrast, patients with diabetes (AOR 0.79) or osteoporosis (AOR 0.86) were less likely to have an irregular pattern of care. Clinic-level number of patients with irregular care, compared with the predicted number, ranged from 0.36 (fewer patients with temporally irregular care) to 1.71 (more patients).

**Conclusions:**

Some patient characteristics are associated with more or less temporally regular patterns of primary care visits. Clinics vary widely on the number of patients with a temporally irregular pattern of care, after adjusting for patient characteristics. Health systems can use the patient-level model to identify patients at high risk for temporally irregular patterns of primary care. The next step is to examine which strategies are employed by clinics that achieve the most temporally regular care, since these strategies may be possible to emulate elsewhere.

## Background

Primary care is thought to have a central role inpromoting better health and forestalling preventable medical problems [1]. A vast literature has shown that the performance of national health systems is tightly linked to the number of primary care providers per population, as well as the extent to which primary care is put at the center of the health system [2–6]. For primary care to be fully empowered to create such value and benefit for patients, there must be opportunities to deliver proactive care which anticipates and prevents problems rather than merely reacting to them after they occur.

**Fig. 1:**
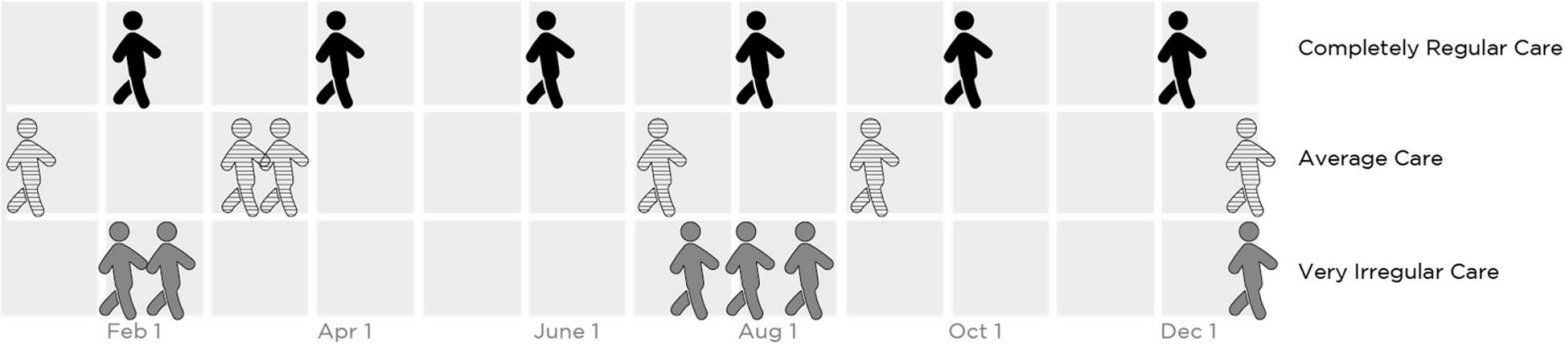
Three example patients, all of whom have six primary care visits in a year. The patients have different levels of temporal regularity of these visits: completely regular care, average care, and very irregular care

For decades, it has been considered generally sound advice to “see your doctor regularly” – particularly for those with chronic health conditions. Implicit in this is the idea that regular visits to the doctor will allow opportunities for high-value proactive and preventive care, as opposed to merely reacting to the “tyranny of the urgent” [1]. For example, three patients with chronic conditions may each have six visits to the primary care doctor in a year – that is, the same absolute frequency of visits. However, one of these patients may visit precisely every 60 days, while a second may visit at somewhat less regular intervals, and a third at extremely irregular intervals (Fig. 1). We would say that the patient with more regular visits has more temporally-regular care.

TR can be thought of as a separate dimension of the idea of continuity of care (COC), which has usually been conceptualized as seeing the same doctor as much as possible, as opposed to a different doctor. A large literature has shown that patients who see the same doctor most or all of the time, presumably their primary care doctor, fare better than those who see different doctors each time [7–15]. This has led some health systems to enact systems to promote higher COC by encouraging patients to see their own doctor as often as possible, as opposed to others. TR can be seen as a separate sort of continuity of care – continuity not with a provider, but measured in time. In effect, this splits the usual advice into two – “see your doctor” (as opposed to a different doctor), “regularly” (as opposed to less regularly).

In recent years, several studies have examined the impact of TR on patient outcomes [16–23]. These studies have generally shown a modest but consistent benefit for patient outcomes from more temporally regular primary care appointments. This benefit was greatest among patients with significant chronic conditions and a high burden of disease. Benefits have included fewer hospitalizations, reduced mortality, and lower costs of care, although different studies found benefits of different magnitudes.

For example, two studies of the same population of older adults (age 65 +) in Australia focused on subgroups with different defining conditions. In the study of patients with epilepsy, compared to the least-regular care, the more-regular quartiles had adjusted hazard ratios (HR) for mortality of 0.62, 0.37, and 0.42 [16]. Reduced mortality with more-regular care was also seen among patients with previous hospitalization for ischemic heart disease, but the effects were more modest (adjusted HR 0.76, 0.71, and 0.71) [17]. It may be that the benefit of more-regular care is stronger among patients with some chronic conditions than others.

Studies have also differed regarding thresholds for TR, namely, whether it matters to have a low TR vs. all others, or whether it matters most to have a high TR, or whether TR has an effect across its entire distribution. Another unanswered question is how much TR is within the control of the health system to remediate, as opposed to merely being a reflection of more difficult or higher-risk patients. The answers to these questions will be important for determining whether health systems should pursue systems to increase low TR, or rather simply use it as marker of patients at elevated risk for complications – and at what level of TR to intervene.

Another issue, about which even less is known, is which patients are most likely to have low TR. Whether TR is ultimately useful as a measure of system-level performance, or as a measure of patient-level risk (or both), it will be useful to know who is most likely to have low TR and therefore be in need of such intervention. In addition, it is important to examine whether TR varies by site of care, after accounting for patient-level differences. This can help inform the question of whether it could be within the system’s control to increase TR.

Here, we used data from Leumit Health Services (LHS), a health maintenance organization (HMO) in Israel, to examine these questions. The study had two aims. First, we aimed to build a model to predict which patients will higher or lower TR, based on patient characteristics. Second, we aimed to examine how TR varies by clinic, after adjusting for patient characteristics.

## Methods

### Data source

LHS is one of four HMOs in Israel; by law, all citizens or permanent residents of Israel belong to one of these HMOs [24]. While switching HMOs is allowed, very few patients do so each year, meaning that the population ensured and cared for by an HMO is extremely stable from year to year [25–29]. The LHS computerized database includes demographics, diagnosis codes, dates of service for inpatient and outpatient care, laboratory values, radiology reports, and prescriptions for medication [25–29]. LHS is the smallest of the four HMOs in Israel, with approximately 700,000 persons covered. The population insured by LHS is generally similar to the population of Israel in many ways, although LHS has a somewhat higher proportion of insured from economically disadvantaged backgrounds than the general population [30].

Because the need for temporally regular primary care for adults without chronic conditions is unproven and even controversial [31], we focused our study on adult patients (age 40 +) with at least one of the following three chronic conditions: heart failure (HF), chronic obstructive pulmonary disease (COPD), or diabetes. We chose these conditions because they are fairly common and have a meaningful impact on patients’ lives and their use of health services. Our data spanned the years 2016–2019, a four-year period. The results presented in this paper are based on TR as calculated for the two-year period 2018–2019. Patient-level variables were determined based on data from 2015, the year prior to our study period.

In addition to the patient being age 40 + and having at least one of the three chronic conditions we studied, we had several other inclusion criteria. Patients were needed to have at least three primary care visits during 2018–2019, because it is difficult to consider the concept of regular primary care unless a certain minimal amount is received. We investigated whether TR could also be characterized based on a one-year period of data, and found that too many patients had fewer than 3 visits and would be excluded (data available upon request). Therefore, we concluded that analyses of TR must be based on at least 2 years of data, and we used the most recent two years of data in our sample for this analysis.

Finally, we excluded all patients who received hemodialysis, even once, during 2015–2019. Patients receiving hemodialysis are evaluated by a physician very frequently, usually at least once a week, during dialysis. Therefore, they receive extremely regular and frequent care, and the concept of TR may not apply to them.

Our study was approved by the research ethics committee of LHS.

### Defining chronic conditions

The thoughtful use of diagnosis codes is a hallmark of high-quality research based on secondary data [32–34]. The list of diagnosis codes that we used to define chronic conditions for this study is found in Table 1. The choice of codes that we used was based on previous research studies using LHS data [25–29]. The LHS database uses International Classification of Diseases, Clinical Modification, version 9 (ICD-9) codes for physical health conditions, and ICD-10 codes for mental health conditions. We defined health conditions based on at least one appearance of any of the codes on the list during the study period (2016–2019). These methods were used to identify the three chronic conditions that were the basis for study eligibility (HF, COPD, diabetes mellitus), as well as the other patient-level comorbid conditions that we used as predictors of TR in our model.

**Table 1:** Diagnosis codes used to define chronic health conditions

Physical Health Conditions	ICD-9 Codes
Atrial Fibrillation	427.3x
Cancer	140–239, **BUT NOT**: 173, 290.40–209.9x, 210–224, 226–229, 232
Chronic Lung Disease^a^	491.x, 492.x, 494.x, 495.x, 496.x, 500–505
Coronary Artery Disease, Angina	413.x
Coronary Artery Disease, History of Myocardial Infarction	412.x
Dementia or Pre-Dementia	331.x
Diabetes Mellitus^a^	250.x, 357.2, 362.0, 366.41
Epilepsy	345.x
Heart Failure^a^	398.91, 402.01, 402.11, 402.91, 404.01, 404.03, 404.11, 404.13, 404.91, 404.93, 425.x, 428.x
Hypertension	401.x, 402.x, 403.x, 404.x, 405.x
Inflammatory Bowel Diseases	555.x, 556.x
Osteoporosis	733.0x
Peripheral Arterial Disease	440.x, 441–442, 443.89, 443.9
Rheumatoid Arthritis	714.0
Sleep Disorders	327.x **BUT NOT** 327.35
History of Stroke	438.x
Venous Thromboembolism	415.x, 453.x
Mental Health Conditions	ICD-10 Codes
Alcohol Misuse	F10.x **BUT NOT** F10.11, F10.21
Anxiety Disorders	F41.x
Attention Deficit Hyperactivity Disorder	F90.x
Bipolar Disorder	F31.x
Depression	F32.x, F33.x
Post Traumatic Stress Disorder	F43.1
Schizophrenia	F20.x, F25.x

^a^Having at least one of these conditions was an inclusion criterion for being in the study

*ICD-9* International Classification of Diseases Code, Clinical Modification, Version 9, *ICD-10* International Classification of Diseases Code, Clinical Modification, Version 10

### Identifying primary care visits

Because this is a study of temporal regularity of primary care visits, an important task was to identify which visits counted as “primary care visits”. In LHS data, all adult primary care is delivered by family physicians, who are clearly identified in the database. However, it has become increasingly common for doctors to deliver care via video-link, telephone, or asymmetric communication (i.e., text messages), as well as in person. For any of these modalities, the doctor records a note for the encounter in the electronic medical record. We used the duration of time the doctor had the patient’s chart open as a measure of the duration of the visit and the complexity of the care that was delivered. We considered a visit of 5 min or more, roughly corresponding to the mean visit duration in our dataset, to constitute a “significant” encounter with the primary care doctor. Shorter visits most likely represented briefer requests, such as requests to refill prescriptions, and did not allow an adequate opportunity to deliver proactive or comprehensive care. This figure of 5 min as a minimal duration for a meaningful primary care visit was informed by the experience of two of the study authors (AJR and YM), who have worked as primary care physicians.

### Dependent variable: patient-level temporal regularity of care

For this study, patient-level TR was the dependent variable – our model predicted each patient’s TR based on patient-level characteristics. TR is measured using the coefficient of variation (COV) for the interval between primary care visits. COV is a measure of statistical variation which is calculated by dividing the standard deviation of the visit interval by its mean [35]. Because it is a standardized figure, COV allows comparisons among patients with different absolute frequencies of primary care visits. Figure 1 shows an example of three patients with a similar frequency of primary care visits (six visits in a year) but extremely different TR. Because of how COV is constructed, a higher COV actually means less temporally regular care. Throughout this manuscript, for clarity, we refer to more- and less-regular care, to prevent confusion.

We investigated the distribution of TR and found that it was not normally distributed. Specifically, we evaluated the normality of TR using the Q-Q plot and the Jarque–Bera test, which is not sensitive to the size of the database [36]. These tests demonstrated that the distribution of TR was clearly not normal. We then log-transformed TR and repeated these tests, but again, the distribution was clearly non-normal. Because TR was not normally distributed, we were unable to predict it using a linear regression model. We therefore dichotomized TR into those patients in the least temporally regular quintile vs. all others, and used a logistic model to predict which patients would be in the least-regular group.

### Independent variables: patient-level characteristics

Patient-level predictors of TR included sociodemographic data and comorbid conditions. Above, we discuss our approach to identifying comorbid conditions using diagnosis codes. The list of chronic conditions that we used as predictors, and the codes we used to define them, appear in Table 1.

Chronic kidney disease was not identified based on diagnosis codes, because we were able to assess it directly using eGFR (estimated GFR). Each patient’s eGFR was characterized based on the lowest value recorded during the years 2018–2019, and was divided into the following levels: 60 + (intact kidney function), 45–59 (mild impairment), 30–44 (moderate impairment), and < 30 (severe impairment). When no information was available to inform eGFR during 2018–2019, we used information from 2016–2017 (2.9% of patients). When no information was available in either period (1.1% of patients), we tried different methods of imputing missing eGFR, including imputing the mean, imputing the mode, and multiple imputation. These choices did not impact the results of our models, so we imputed the mode (60 + , or intact kidney function).

Although diagnosis codes do exist for cigarette smoking, we instead used LHS’ direct records of who is a current smoker, which is recorded separately. This captured many times as many smokers as the codes would have captured. Former smokers and current smokers are recorded separately by LHS, so the patients with this variable were current smokers at the time of the study period.

Sociodemographic data are as follows. Age was based on the year of birth and was divided into the following categories: 40–49, 50–59, 60–69, 70–79, 80–89, and 90 + . Sex is reported as male or female. Ethnic/social groups included Arabs, Ultra-Orthodox Jews, and the “General Population” (all others). Patients were characterized as living in one of four regions of Israel: Central (including Tel Aviv), Jerusalem and its surroundings, the South, and the North. Area socioeconomic status (SES) was provided by Points Location Intelligence [37] and is divided into ten levels, from the poorest (1) to the wealthiest (10). We grouped SES into four groups: 1–3 (poorest), 4–5, 6–7, and 8–10 (wealthiest).

### Assigning each patient to a primary clinic

Because we planned to examine clinic-level variation in case mix adjusted TR, we needed to assign each patient to a primary clinic. Case mix adjustment is an approach for examining variations in performance by provider or by site of care, after adjusting for differences in the patient population seen by each provider or site. After such adjustment, any remaining differences should be attributable to differences in the care being provided, and not to having harder or easier patients [38]. Each patient is already assigned to a primary clinic in the LHS data, based on his or her selection of a primary care doctor. The LHS database contained 330 clinics in total.

### Statistical analyses

Analyses were divided into patient-level analyses and clinic-level analyses. We began our patient-level analyses with bivariate analyses, examining the effect of each independent variable on patient-level TR. We used a logistic regression model to examine the odds ratio for the patient to be in the quintile with the least temporally regular care, as opposed to the other 80% of the population, first without control variables, and then with them in a fully adjusted model.

We then used our fully adjusted model to predict a least-regular pattern of care to examine differences among the 239 LHS clinics in our sample with at least 30 patients meeting our study criteria. Clinics with fewer than 30 patients (*n* = 91) were excluded from this stage of the analysis, because of concern about the stability of estimates with smaller numbers of patients. We used our logistic model to predict the expected number of patients at each clinic who would be in the least temporally regular fifth of the sample, based on patient characteristics. We then compared this to the observed number of patients in this category, using an observed divided by expected approach (O/E). An approach comparing O to E is often used in the case-mix adjustment literature [38–40]. An O/E score of 1 means that the site is performing as might be expected, while a score less than 1 means that the site had fewer patients receiving irregular care than expected, and a score more than 1 means the reverse.

All statistical analyses were performed using the R statistical package, version 4.1.2.

## Results

### Patient characteristics

Our study included 70,095 patients who were age 40 + , had one of three selected chronic conditions (HF, COPD, or diabetes), and had at least 3 primary care visits during 2018–2019, which was sufficient to allow us to calculate TR. Patient characteristics are shown in Table 2. A majority of the sample (59%) was between ages 50–69. The sample was evenly divided between males and females. A majority of the sample (59%) was of lower or lowest SES. As would be expected of an older sample, all of whom had at least one of the three selected chronic conditions, there was a relatively high rate of other chronic conditions. For example, 11% of patients had experienced a myocardial infarction in the past, 21% had osteoporosis, and 21% had some degree of impaired kidney function. There was also a relatively high burden of mental health conditions, such as a 28% prevalence of depression.

**Table 2:** Baseline characteristics of the included patients (*n* = 70,095)

Variables	Cell count	Percent
Age Groups (year born)		
40–49 (1966–1975)	8,890	13
50–59 (1956–1965)	18,333	26
60–69 (1946–1955)	22,831	33
70–79 (1936–1945)	13,424	19
80–89 (1926–1935)	5,907	8.4
90 + (1910–1925)	710	1.0
Sex		
Male	35,066	50
Female	35,029	50
Ethnic/cultural group		
Arabs	11,345	16
Ultra-Orthodox Jews	4,166	5.9
Others	53,959	77
Region of Israel		
North	18,234	26
Central	21,234	30
Jerusalem	9,196	13
South	21,431	31
Socioeconomic Status (SES)		
Highest	5,821	8.0
Higher	11,263	31
Lower	30,285	43
Lowest	11,263	16
PHYSICAL HEALTH CONDITIONS		
Atrial Fibrillation	8,548	12
Cancer	7,693	11
Chronic Lung Disease	20,279	29
Coronary Artery Disease, Angina	20,270	29
Coronary Artery Disease, MI	7,612	11
Dementia/Pre-Dementia	820	1.2
Diabetes	54,899	78
Epilepsy	643	0.9
Headache	4,671	7.0
HF	11,761	17
Hypertension	32,414	46
Inflammatory Bowel Disease	1,233	1.8
Osteoporosis	14,385	21
Peripheral Arterial Disease	7,572	11
Rheumatoid Arthritis	1,965	2.8
Smoking, Current	13,672	20
History of Stroke	3,302	5.0
Venous Thromboembolism	3,709	5.0
eGFR (lowest one recorded)		
60 +	55,294	79
45–59	8,056	11
30–44	4,205	6.0
< 30	2,540	4.0
MENTAL HEALTH CONDITIONS		
Alcohol Misuse	215	0.3
ADHD	1,272	1.8
Anxiety Disorders	20,860	30
Bipolar Disorder	998	1.4
Depression	19,789	28
PTSD	1,795	2.6
Schizophrenia	1,353	1.9

### Patient-Level predictors of temporally irregular care

We characterized each patient on TR. The TR score ranged from 0.00 (total temporal regularity; precisely the same number of days between visits) to a high of 3.57. The median TR was 0.93 and the mean was 0.95 (SD 0.32). For our models, we defined a group with TR 1.2 or higher, corresponding to the quintile of patients with the least-regular pattern of care.

Table 3 shows the patient-level predictors of being in the least temporally regular quintile. All differences discussed below are statistically significant. Compared to the youngest group (age 40–49), older patients were less likely to be in the least-regular group. For example, age 70–79 had an adjusted odds ratio (AOR) of 0.83 compared to the reference category. Males were more likely to be in the least temporally regular quintile, compared to females (AOR 1.19). Members of minority groups were also more likely to be in the least temporally regular quintile, compared to the general population (AOR 1.10 for Ultra-Orthodox Jews, 1.19 for Arab). Area-level SES was not a significant predictor of regularity of care, after adjusting for other factors. Some health conditions were associated with a higher or lower odds of being in the least temporally regular quintile, compared to not having the condition. Patients with diabetes (AOR 0.79), cancer (AOR 0.85) or osteoporosis (AOR 0.86) were among the least likely to have an irregular pattern of care. Patients with a history of myocardial infarction (AOR = 1.07), atrial fibrillation (AOR = 1.08), and current smokers (AOR = 1.12) were among the most likely to have an irregular pattern of care. Patients with mental health diagnoses were less likely to be in the least-regular group, particularly those with schizophrenia (AOR = 0.80). Impaired kidney function did not predict temporal regularity, after controlling for other variables. The c-statistic for the entire predictive model was 0.58, indicating a modest level of prediction.

**Table 3:** Bivariate and multivariable logistic models predicting temporal regularity (TR) as a binary outcome. The model predicts which patients are more likely to be in the quintile with the least temporally regular pattern of primary care (TR ≥ 1.2). Values greater than 1 mean that a patient is more likely to be in the group with a less regular pattern of care. The multivariable model is adjusted for all the variables in the table

Variables	OR unadjusted bivariate model	CI unadjusted	OR adjusted model	CI adjusted
Age Groups (year born)				
40–49 (1966–1975)	REF		REF	
50–59 (1956–1965)	0.88 †	0.82, 0.93	0.92 *	0.87, 0.98
60–69 (1946–1955)	0.74 †	0.70, 0.79	0.86 †	0.81, 0.91
70–79 (1936–1945)	0.67 †	0.62, 0.71	0.83 †	0.77, 0.89
80–89 (1926–1935)	0.66 †	0.61, 0.72	0.86 *	0.78, 0.94
90 + (1910–1925)	0.72 *	0.59, 0.87	0.90	0.73, 1.10
Sex				
Male	1.31 †	1.26, 1.36	1.19 †	1.14,1.24
Female	REF		REF	
Ethnic/cultural group				
Arabs	1.29 †	1.23, 1.36	1.19 †	1.12, 1.27
Ultra-Orthodox Jews	1.18 †	1.09, 1.27	1.10 *	1.01, 1.12
Others	REF		REF	
Region				
North	1.14 †	1.08, 1.20	1.04	0.98, 1.10
Central	REF		REF	
Jerusalem	1.16 †	1.09, 1.23	1.10 *	1.03, 1.17
South	1.00	0.96, 1.06	1.00	0.95, 1.06
SES				
Highest	REF		REF	
Higher	1.10 *	1.02, 1.19	1.09 *	1.01, 1.17
Lower	1.16 †	1.08, 1.25	1.07	0.99, 1.16
Lowest	1.29 †	1.19, 1.40	1.06	0.96, 1.17
PHYSICAL CONDITIONS				
Atrial Fibrillation	0.95	0.91, 1.01	1.08 *	1.01, 1.15
Cancer	0.77 †	0.72, 0.82	0.85 †	0.80, 0.91
Chronic Lung Disease	1.04 *	1.00, 1.09	0.93 *	0.88, 0.98
Coronary Artery Disease, Angina	0.85 †	0.82, 0.89	0.90 †	0.86, 0.94
Coronary Artery Disease, MI	1.08 *	1.02, 1.15	1.07 *	1.01, 1.14
Dementia/Pre-Dementia	0.90	0.75, 1.08	1.05	0.87, 1.26
Diabetes	0.83 †	0.80, 0.87	0.79 †	0.75, 0.84
Epilepsy	0.89	0.72, 1.08	0.94	0.76, 1.15
Heart Failure	0.96	0.92, 1.01	0.98	0.92, 1.04
Hypertension	0.81 †	0.78, 0.84	0.92 †	0.88, 0.96
Inflammatory Bowel Disease	0.95	0.82, 1.10	1.00	0.86, 1.15
Osteoporosis	0.71 †	0.67, 0.74	0.85 †	0.81, 0.90
Peripheral Arterial Disease	0.88 †	0.82, 0.93	0.92 *	0.86, 0.99
Rheumatoid Arthritis	0.83 *	0.73, 0.93	0.92	0.81, 1.03
Smoking, Current	1.26 †	1.20, 1.32	1.12 †	1.06, 1.18
History of Stroke	0.88 *	0.80, 0.96	0.94	0.85, 1.03
Venous Thromboembolism	1.01	0.92, 1.09	1.09	1.00, 1.18
eGFR (lowest one recorded)				
60 +	REF		REF	
45–59	0.85 †	0.80, 0.91	0.98	0.91, 1.04
30–44	0.85 †	0.78, 0.92	1.00	0.91, 1.09
< 30	0.86 *	0.78, 0.96	1.00	0.89, 1.11
MENTAL HEALTH CONDITIONS				
Alcohol Misuse	1.48 *	1.08, 2.00	1.28	0.94, 1.74
ADHD	0.98	0.85, 1.13	0.91	0.79, 1.04
Anxiety Disorders	0.83 †	0.80, 0.87	0.91 †	0.87, 0.95
Bipolar Disorder	1.01	0.86, 1.18	1.12	0.95, 1.31
Depression	0.82 †	0.79, 0.86	0.92 †	0.88, 0.96
PTSD	0.96	0.85, 1.09	0.97	0.85, 1.09
Schizophrenia	0.88	0.76, 1.01	0.80 *	0.69, 0.93

^*^ *p* < 0.05

^†^ *p* < 0.001

### Clinic-Level patterns of temporal regularity

**Fig. 2:**
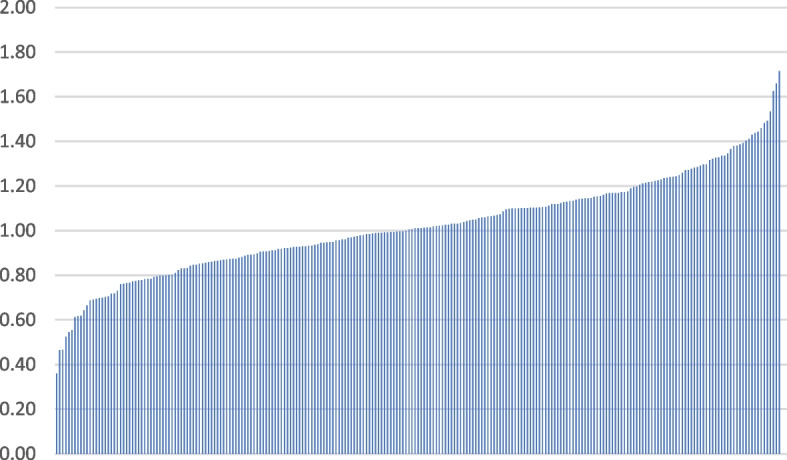
Variation in observed/expected score among 239 Leumit clinics with at least 30 patients meeting our study criteria. A score of 1.0 means that the clinic had precisely as many patients in the least-regular group as would be predicted based on case mix. A score above 1 means that the clinic had more patients than expected who received temporally irregular care, and a score below 1 means the reverse

We then examined differences among the 239 LHS clinics in our sample. Each clinic had between 33–3160 patients under management that met our study criteria. We used our logistic model to predict the expected number of patients at each clinic who would be in the least temporally regular fifth of the sample, based on patient characteristics. We then compared this to the observed number of patients in this category, using and observed divided by expected approach (O/E). Figure 2 shows all the clinics and their O/E scores. O/E scores ranged from a low of 0.36 to a high of 1.71. Extreme O/E scores were not only recorded by clinics with smaller populations; some of the larger clinics also recorded extreme scores. There were 93 clinics with a score between 0.90–1.10 (or, similar to what might be predicted), constituting 39% of the sample. This means that 61% of the clinics had either considerably fewer patients with irregular patterns of care, or considerably more patients, than might be expected based on patient characteristics alone.

## Discussion

In this study, we examined patient-level predictors of having a highly irregular pattern of primary care. We used this model to examine clinic-level variation in TR, specifically among patients over age 40 with one of three chronic conditions, after adjusting for patient-level characteristics. We found that among 239 clinics in a single HMO, the number of patients with a highly irregular pattern of care varied from about a third as much as expected to almost twice as much as expected. This suggests that clinic-level practices may play a role in contributing to TR, and that it could be within the power of the health system to encourage a more temporally regular pattern of care. It would therefore seem worthwhile to further investigate potential causes of the between-clinic differences we observed here.

A growing literature has shown that a temporally regular pattern of primary care is associated with better patient outcomes in later years, likely representing the benefit of proactive care that was delivered earlier [16–18, 23]. However, there are significant unknowns about the concept of temporal regularity (TR). Among them, there have been no previous studies of which patient-level characteristics are associated with more- or less-regular patterns of care. In addition, little is known about how much TR varies among clinics, after adjusting for patient characteristics. Our findings suggest that we may be able to identify some patients who are more likely to have irregular patterns of care. This information could be used to prospectively identify such patients and target them for programs to encourage more regular follow-up and more proactive primary care.

Our findings also suggest that some clinics do a particularly good job of encouraging older patients with serious chronic conditions to follow-up more regularly. This may consist of efforts to bring in patients who have missed their appointments, or some other kind of outreach. It is noteworthy that the variation that we observed is occurring within a single HMO, where all the clinics share a single medical record and a single infrastructure supporting the clinics. In an ongoing study, we are using qualitative methods to examine what distinguishes clinics with a pattern of more temporally-regular care than predicted from clinics with a pattern of less-regular care. This, in turn, may allow health systems to try to identify and promote best practices that can support more temporally-regular follow-up, along with the accompanying benefits to patient health.

While there have been a number of studies about the impact of patient-level TR on patient outcomes [7–15], there has only been one previous study that examined patient-level predictors of TR and site-level variation in TR [23]. In that study, we examined a group of safety-net clinics, which mostly serve poor and underserved patients. Patient-level predictors of a more-regular pattern of care, in that study, were sometimes similar to our findings here, and sometimes different. However, the most important similarity between the two studies, in terms of findings, was that clinics varied considerably on TR, even after adjusting for patient-level characteristics. This variation across clinics implies that it may be possible to design programs to increase the temporal regularity of primary care visits among patients with chronic conditions. First, it would be necessary to better understand some of the factors contributing to this variation across clinics.

Indeed, our understanding of how care is delivered in the LHS system would already support some hypotheses for what is underlying this between-clinic variation in TR. In particular, LHS has ongoing initiatives to coordinate care for certain chronic conditions, including the three chronic conditions we studied here (diabetes mellitus, COPD, and heart failure) and also some others (e.g., cancer, autism spectrum disorders) [41, 42]. The goal of these initiatives is to help improve quality of care for these conditions, in many cases aiming for benchmarks that are set by Israel’s national set of quality measures, the Quality Indicators in Community Healthcare (QICH) measure set [43]. These LHS care coordination initiatives are built into the LHS electronic medical record and are supported by frequent events for providers and other kinds of administrative support [26, 27]. The LHS initiative to support care for diabetes mellitus is particularly strong and longstanding [25, 44], and there are a large number of quality measures for patients with diabetes that may encourage more regular contact with the health system [43]. This may have contributed to our finding of more temporally regular visits among patients with diabetes mellitus. In fact, it could be that such disease management programs could potentially play an important role in increasing TR in the future for other groups of patients. We are examining this, and other hypotheses, in our ongoing study.

The present study has important strengths, including that it uses patients from a large and integrated HMO and that we had considerable clinical detail in our dataset. It is also a strength that efforts to study TR encompass several different settings, including Australia, the United States, and now Israel. However, we also acknowledge important limitations. Foremost among them is that our study suggests that social context may have a lot to do with temporal patterns of care and with who chooses to follow-up regularly. In addition, we were unable to observe behavioral choices such as patients who choose not to follow-up when requested to do so. For this reason, we plan a follow-up qualitative study, to examine these issues in greater detail that is possible with a large secondary dataset such as we used here. Another limitation that we should mention is that we only studied one of the four Israeli HMOs. However, we would strongly expect to find that the other HMOs also vary on TR at the clinic level. While future studies should examine TR and its variation in different health systems, our experience so far has suggested that TR varies in every health system. Another potential limitation is that it required two years of data to characterize TR, with enough visits to populate the variable, without losing too many patients due to not having enough visits. This could complicate efforts to measure TR on an ongoing basis, since most quality measures are followed on an annual basis. Finally, although we presented our study using terms such as “prediction”, which is customary in the case-mix adjustment literature, we do acknowledge that this is a cross-sectional study design, which complicates assignment of causality [37–39]. However, it is worth noting that all case-mix adjustment efforts use cross-sectional data, and that it is standard to refer to concepts such as “predictors” in the context of case mix adjustment [37–39].

## Conclusions

We examined the issue of temporal regularity of primary care using a large database from an Israeli HMO. We found considerable clinic-level variation on TR, even after adjusting for patient-level characteristics. This implies that the health system could potentially have a role in promoting more-regular patterns of follow-up. In a future study, we plan to examine this issue more closely, using qualitative methods, to characterize what practices are common to the sites that achieve more temporally regular care.

## Data Availability

LHS data may be made available to researchers who partner with an LHS researcher, and who apply for data access. Statistical code is available upon request.

## Abbreviations

COC: Continuity of Care
COPD: Chronic Obstructive Pulmonary Disease
COV: Coefficient of Variation
eGFR: Estimated Glomerular Filtration Rate
HF: Heart Failure
HMO: Health Maintenance Organization
ICD-9 CM: International Classification of Diseases, Clinical Modification, Verison 9
ICD-10 CM: International Classification of Diseases, Clinical Modification, Verison 10
SES: Socioeconomic Status
TR: Temporal Regularity
